# Tetrahydrocannabinol (Delta 9-THC) Treatment in Chronic Central Neuropathic Pain and Fibromyalgia Patients: Results of a Multicenter Survey

**DOI:** 10.1155/2009/827290

**Published:** 2009-10-25

**Authors:** Janet Weber, Marcus Schley, Matthias Casutt, Helmut Gerber, Guido Schuepfer, Roman Rukwied, Wolfgang Schleinzer, Michael Ueberall, Christoph Konrad

**Affiliations:** ^1^Department of Anesthesiology, Intensive Care, Emergency Medicine and Pain Therapy, Kantonsspital Lucerne, 6000 Lucerne, Switzerland; ^2^Department of Anesthesiology, Medical Faculty Mannheim, University of Heidelberg, 68167 Mannheim, Germany; ^3^Department of Anesthesiology and Pain Medicine, Swiss Paraplegic Center, 6207 Nottwil, Switzerland; ^4^Department of Neuroscience, Algesiology and Pediatrics, 90419 Nuremberg, Germany

## Abstract

Central neuropathic pain is difficult to treat, but delta 9-Tetrahydrocannabinol (delta 9-THC) may be a promising therapeutic agent. We administered in 172 patients on average 7.5 mg delta 9-THC over 7 months. Of these, 48 patients prematurely withdrew due to side effects, insufficient analgesia, or expense of therapy. Thus, 124 patients were assessed retrospectively in a multicenter telephone survey. Reported changes in pain intensity, recorded on a numeric rating scale (NRS), Pain Disability Index (PDI), Medical Outcomes Short-Form (SF-12), Quality of Life Impairment by Pain (QLIP), Hospital Anxiety Depression Scale (HADS), and amount of concomitant pain medication were recorded. Psychometric parameters (PDI, SF-12, QLIP, HADS) and pain intensity improved significantly during delta 9-THC treatment. Opioid doses were reduced and patients perceived THC therapy as effective with tolerable side effects. About 25% of the patients, however, did not tolerate the treatment. Therapy success and tolerance can be assessed by a transient delta 9-THC titration and its maintained administration for several weeks. The present survey demonstrates its ameliorating potential for the treatment of chronic pain in central neuropathy and fibromyalgia. A supplemental delta 9-THC treatment as part of a broader pain management plan therefore may represent a promising coanalgesic therapeutic option.

## 1. Introduction

Various drugs belonging to different pharmacologic classes are currently prescribed and administered for the treatment of central neuropathic pain and fibromyalgia syndrome, including antidepressants, first- and second-generation anticonvulsants, antiarrhythmics, topical agents, N-methyl-D-aspartate receptor antagonists, and opioid analgesics [1, 2]. The long-term use of these drugs is often limited by adverse effects or patient's compliance. About 25% of the patients consulting pain clinics suffer from neuropathic pain states [3]; however, central neuropathic pain is often refractory to many treatments.

 Over the last decades, the cannabinoids and their chemistry, the enzymes and receptors involved in their metabolism, as well as their assumed physiological and pathological roles in human pain pathways, have been characterized in detail [4–8].

 Among hundreds of constituents of *cannabis sativa*, delta 9-Tetrahydrocannabinol (delta 9-THC) is the main active constituent. It is one of the natural compounds of marijuana (cannabis, hash) and was first synthesized by Mechoulam in 1967 [9]. The therapeutic use of cannabis has been widely reviewed [10–14] but the clinical use so far has been conflicting and is limited partly due to the narrow therapeutic index of delta 9-THC [15]. Several practical problems as well as ethical issues due to their potential abuse have been raised; however, delta 9-THC meanwhile is legally available for medical treatment throughout the world (US, Europe, Africa). It was reported that THC revealed beneficial effects on pain and spasticity caused by multiple sclerosis [16, 17]. Also, THC might be effective in the treatment of chemotherapy-induced emesis, depression, low appetite, paresthesia, muscle pain, and painful neuropathy [18, 19], and cannabidiol represents a promising therapeutic agent in neurodegenerative disorders [10]. The indication for a cannabinoid-based therapy, however, demands a thorough risk/benefit evaluation. Particularly the patients' history of substance abuse has to be considered [20], as well as the specific diagnosis of the patients disease (e.g., cancer, HIV, MS, etc.). The efficacy of cannabinoids has been reported to be controversial in the literature when neuropathic pain [21, 22], experimentally induced pain [23], or hyperalgesia [24] are concerned. Therefore, delta 9-THC should be classified as a coanalgesic [25, 26] rather than pure analgesic drug.

 Here, we explored the clinical applicability and efficacy of delta 9-THC administration supplemental to existing medication plan of patients suffering from refractory central neuropathic pain and fibromyalgia by means of a standardized retrospective telephone interview survey.

## 2. Material and Methods

After approval by the Ethics Committee II, Medical Faculty Mannheim, University of Heidelberg, 19 practitioners within different Federal States of Germany recruited 172 patients diagnosed with central neuropathic pain and fibromyalgia. 

 The 19 medical specialists were particularly experienced in anaesthesiology, neurology, and algesiology, as well as their extensive experience with the therapy of chronic and neuropathic pain patients. None of the 19 practitioners had received previously industry sponsored research money or were in cooperation with the authors. All practitioners had prescribed THC before and at least 10 times per year and were members of the German Association for Pain Therapy (DGS).

 Delta 9-THC is available on prescription only. Inclusion criteria for prescription were primarily ineffectiveness of current pain therapy, but also sleep disturbances and decreased quality of life reported by the patients. For titration of the drug, all practitioners increased the oral delta 9-THC dose weekly by 2.5 mg and as long as no severe side effects were reported. Dosing did not exceed 15 mg per day, unless medication was ineffective. Delta 9-THC was taken in the morning and evening and therapy was maintained for at least 3 months. Each medical specialist contributed on average 5–8 patients to the survey, who signed a written informed consent prior to delta 9-THC medication. Only patients with a central neuropathic pain syndrome or fibromyalgia were included, irrespective of gender, age, or ethnic origin. The existence of central neuropathic pain was defined when patients suffered central neuropathy due to an inflammatory damage or trauma of the central nervous system. Exclusion criteria were chronic neuropathic pain states of peripheral origin. All 172 patients received, in addition to their current medication, delta 9-THC (dronabinol; Delta 9 Pharma, Neumarkt, Germany) for pain therapy. Demographic data, general health, diagnosis, and medical history were compiled from the patients' files. A researcher, who was experienced in performing telephone interviews, but had no association with the authors or the 19 medical specialists, was employed to collect the data. The interviewer was not informed about the medication plan of the patient or the objective of the survey. Following parameters were recorded: patients' self-reported pain intensity (verbal and numeric ratings), efficacy and tolerability of the pre-THC regimen, efficacy and tolerability of delta 9-THC medication, improvement in mood and quality of life, general impairment, and work performance. Efficacy and tolerability of pre-THC regimen and current analgesic therapy was recorded using a rating scale in the range from 1 to 6, with the descriptors (1) “very good,” (2) “good,” (3) “satisfying,” (4) “critical,” (5) “insufficient,” and (6) “poorly.” Further, a psychometric assessment was performed, including the Medical Outcomes Short-Form (SF-12), the Pain Disability Index (PDI), the Quality of Life Impairment by Pain (QLIP), and the Hospital Anxiety and Depression Scale (HADS).

 The perceived pain intensity was estimated by the patients on a 6-point Verbal Rating Scale (VRS) with the particular descriptors (corresponding values given in brackets): no pain (0), slight pain (2), moderate pain (4), intense pain (6), very strong pain (8), worst pain imaginable (10). In addition, maximum pain intensity was estimated on an 11-point Numeric Rating Scale (NRS) with the endpoints 0 (no pain) and 10 (maximum pain imaginable). Also, patients were requested to evaluate possible changes of symptoms resulting from delta 9-THC treatment. In particular, characteristics of pain intensity and pain quality, tolerability of delta 9-THC therapy, and changes in coanalgesics were recorded. Patients were instructed to specify side effects by means of a list of thirty characters. Recordings made by the patients were documented independently from the practitioner's assessment regarding adverse effects and effectiveness. 

 Data were analyzed by means of descriptive statistical analysis using SPSS software package (SPSS Inc., Chicago, Illinois, USA). Patients' values prior to delta 9-THC therapy and values during THC treatment were evaluated for significant differences between the values identified by ANOVA. In order to analyze categorical data in which *P*-values were calculated for c (columns) × r (rows), StatExact 5.0 software package (Cytel Inc., Massachusetts, US) was used and Pearson's chi-square test applied (*P* < .05). Data of psychometric assessment are depicted as mean + standard deviation (SD).

## 3. Results

Of the 172 patients, 48 prematurely withdrew within 2 weeks from the survey due to tiredness as side effect (*n* = 6), insufficient therapy effect (*n* = 5), expenses of delta 9-THC therapy (*n* = 29), or other reasons (*n* = 23) that include mainly dizziness and an enhanced appetite. Consequently, complete data sets were recorded from 124 patients only (77 women, 47 men, average age 55 ± 13 years).

### 3.1. Demographic Details and Diagnosis

Most of the patients (*n* = 114) had been suffering from pain for more than three years, other patients for 1 to 3 years (*n* = 7). Of 3 patients, however, no duration of pain history could be obtained.

 The etiology of the patients diagnosed with central neuropathy (*n* = 92) was primarily of inflammatory origin (*n* = 43), such as multiple sclerosis (*n* = 32), encephalitis (*n* = 9), and others (*n* = 2), or due to a trauma of the central nervous system (*n* = 49), such as stroke (*n* = 10), paraplegia (*n* = 8), intracranial injury (*n* = 4), neoplasm (*n* = 4), or others (*n* = 23) (see Table 1). A somatic cause of pain in neoplasm patients due to the location and extent of the neoplasm was excluded by ultrasound and computer tomography examination. 

 In addition to central neuropathy patients, 32 fibromyalgia patients suffering also chronic pain were included in the survey. A controversy is prevailing whether fibromyalgia patients can be included as a central neuropathic pain state. It has been reported in former studies that fibromyalgia is characterized by widespread generalized pain with an abnormal nociceptive central processing [27, 28]. By contrast, most clinicians being involved in the treatment of chronic pain would argue that fibromyalgia is not a purely central neuropathic pain syndrome, as reviewed recently [29]. Therefore, we analysed in the present survey the fibromyalgia patients as independent group from patients diagnosed with central neuropathic pain (see Table 3). Fibromyalgia was diagnosed according to the criteria of the American College of Rheumatology [30], including tender point on the physical examination [31, 32]. All patients complained widespread pain and revealed soreness upon pressure in at least 11 out of 18 tender points.

### 3.2. Analgesic Medication and Its Efficacy prior to Therapy

Patients most commonly received nonopioids, such as NSAID's *n* = 60 (48%), COX2-inhibitors *n* = 34 (27%), paracetamol *n* = 29 (23%), metamizol *n* = 44 (36%) (see Table 2), but also opioids, tramadol *n* = 35 (28%), morphine *n* = 22 (18%), or hydromorphone *n* = 17 (14%), as well as coanalgesics, such as antidepressants *n* = 68 (55%) or anticonvulsants *n* = 40 (32%). 

 The efficacy of the analgesic medication prior to delta 9-THC treatment was assessed by the patients as follows: very good *n* = 1 (0.8%), good *n* = 4 (3%), satisfactory *n* = 15 (12%), sufficient *n* = 24 (19%), insufficient *n* = 58 (47%), poor *n* = 20 (16%).

### 3.3. Analgesic Medication during Delta 9-THC Therapy

During therapy with delta 9-THC, administration of analgesics could be reduced. Only nonopioids were maintained in 36 patients (29%), opioids continued in 39 patients (31%), and coanalgesic medication pursued in 54 patients (43%) (see Table 2).

### 3.4. Delta 9-THC Dose and Duration of Administration

Delta 9-THC was administered as liquid (*n* = 78, 63%), as capsule (*n* = 27, 22%), or both in combination (*n* = 19, 15%). On average, a mean daily dose below 7.5 mg delta 9-THC was administered to 47 patients (38%), dosages between 7.5 and 15 mg received 26 patients (21%), and dosages >15 mg were taken by 16 patients (13%). In 35 patients (28%) the daily taken delta 9-THC dose could not be obtained. Overall, the median administered delta 9-THC concentration was on average 7.5 mg per day (interquartile range 5–12.5 mg). 

 35 patients received delta 9-THC medication up to 4 months (28%), 38 patients 4–24 months (31%), and 16 patients for more than 24 months (13%). No duration recordings, however, were obtained from 35 patients (28%). Thus, on average, delta 9-THC treatment lasted 217 days (interquartile range 27–412 days).

### 3.5. Pain Score

Pain intensity was estimated on a verbal rating scale (VRS), as described previously.

**Figure 1:**
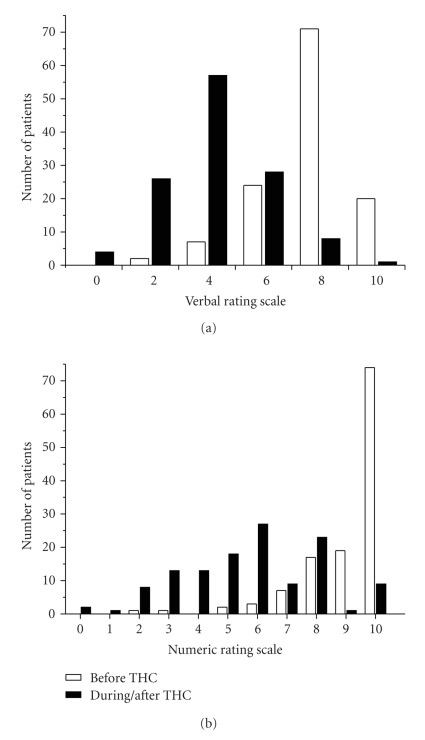
Number of patients and their estimation of the perceived pain intensity before (white bar) and during/after (black bar) delta 9-THC therapy by means of (a) Verbal Rating Scale (VRS) and (b) Numeric Rating Scale (NRS). Values of the VRS indicate “no pain” (0), “slight pain” (2), “moderate pain” (4), “intense pain” (6), “very strong pain” (8), “worst pain imaginable (10). The endpoints of the NRS indicate “no pain” (0) and “worst pain imaginable” (10).

 Prior to delta 9-THC administration, light pain was recorded in *n* = 2 patients (2%), moderate pain in *n* = 7 (6%), intense pain in *n* = 24 (19%), very strong pain in *n* = 71 (57%), and worst pain imaginable in *n* = 20 (16%) (see Figure 1(a)). Following delta 9-THC administration, verbally reported pain intensity improved significantly (*P* < .001, Pearson's chi-square test), revealing a median value of 4 “moderate pain” after delta 9-THC in comparison to a median value of 8 “very strong pain” prior to THC-therapy. In particular, during delta 9-THC administration, no pain was reported on the verbal rating scale in *n* = 4 patients (3%), slight pain in *n* = 26 (21%), moderate pain in *n* = 57 (46%), intense pain in *n* = 28 (23%), very strong pain in *n* = 8 (7%), and worst pain imaginable still in 1 patient (see Figure 1(a)). 

 Subgroup analysis of fibromyalgia revealed no differences of pain intensity to the group of inflammatory- and trauma-evoked central neuropathic pain patients prior to and during/after delta 9-THC medication. Prior to delta 9-THC administration, mean pain intensity (VRS) of fibromyalgia was on average 7.9 ± 1.5, which was reduced to 4.4 ± 1.5 during/after THC-treatment. Similarly, inflammatory pain patients reported a mean pain intensity of on average 7.6 ± 1.7 before THC, and 4.2 ± 1.9 after delta 9-THC therapy. Trauma-induced central neuropathic pain patients estimated pain at 7.6 ± 1.4 prior to and 3.8 + 1.5 during/after delta 9-THC medication (see Table 3).

 Maximum perceived pain intensity was estimated on a numeric rating scale (NRS), as described previously. Pain reported by patients before THC-therapy was NRS <6 in *n* = 4 (3%), NRS 6 in *n* = 3 (2%), NRS 7 in *n* = 7 (6%), NRS 8 in *n* = 17 (14%), NRS 9 in *n* = 19 (15%), and NRS 10 in *n* = 74 (60%). In contrast, maximum pain estimated by the patients after delta 9-THC therapy was perceived at NRS <6 in *n* = 55 patients (44%), NRS 6 in *n* = 27 (22%), NRS 7 in *n* = 9 (7%), NRS 8 in *n* = 23 (19%), NRS 9 in *n* = 1 (0.8%), and NRS 10 in *n* = 9 (7%) (see Figure 1(b)).

 No significant difference of maximum pain was obtained between fibromyalgia syndrome and inflammatory- or trauma-evoked central neuropathic pain patients. On average, maximum pain intensity (NRS) was in fibromyalgia recorded at 9.3 ± 1.1 prior to delta 9-THC and 6.1 ± 2.1 thereafter. Central chronic pain patients reported a maximum pain intensity of 8.7 ± 1.7 (inflammatory pain) and 9.3 + 1 (trauma pain) prior to delta 9-THC, but 4.9 + 2.4 (inflammatory pain) and 5.3 + 1.7 (trauma pain) after THC-therapy, respectively (see Table 3).

### 3.6. Psychometric Assessment

**Figure 2:**
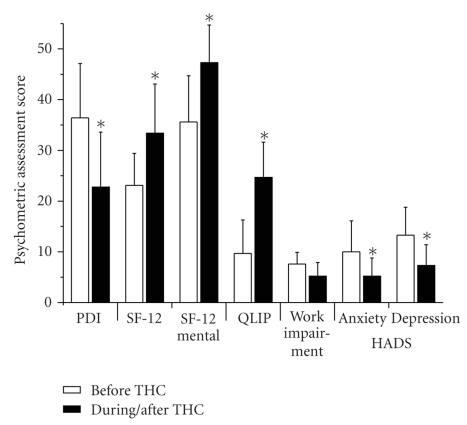
Psychometric assessment of the patients before (white bar) and during/after (black bar) delta 9-THC therapy. Particularly Pain Disability Index (PDI), Quality of Life (QLIP), and Hospital Anxiety Depression Scale (HADS) improved significantly in response to delta 9-THC treatment.

Prior to delta 9-THC administration, the Pain Disability Index (PDI) was on average 36.4 ± 10.7, which improved significantly to 22.8 ± 10.8 with the delta 9-THC administration (*P* < .001). Similarly, improved Medical Outcomes Short-Form scores (SF-12) were recorded, with significantly increased health-related subscale scores from 23.1 ± 6.3 before therapy to 33.4 ± 9.7 after delta 9-THC administration (*P* < .001) and a comparable increase of the mental subscale score from 35.6 ± 9.1 to 47.3 ± 7.4 (*P* < .001), respectively. Quality of life assessed by the pain summary scale (QLIP) also improved by about 150% from 9.7 + 6.6 before therapy to 24.7 + 6.9 after delta 9-THC (see Figure 2). In addition, pain-related disability of patients to perform their daily professional work reduced from a mean score impairment of 7.6 ± 2.3 prior to therapy to 5.2 ± 2.7 after delta 9-THC. Finally, mean Hospital Anxiety and Depression Scale (HADS) was attenuated for anxiety from 10 ± 6.1 to 5.2 ± 3.6 and for depression from 13.3 ± 5.5 to 7.3 ± 4.1, respectively (*P* < .001).

 The vast majority of patients (92%) evaluated the delta 9-THC therapy as efficient and accepted its administration as coanalgesic. In contrast, no improvement was reported in 3% of the patients, and 5% complained of increased pain (data not shown).

### 3.7. Adverse Effects

In 12 patients (10%), adverse effects were reported but tolerated during delta 9-THC therapy, of which tiredness (*n* = 3) and sedation or dizziness (*n* = 4) were primary side effects.

## 4. Discussion

Cannabis (delta 9-THC) has been recognized as appetite stimulant and antiemetic drug, and therefore had been administered clinically to ameliorate side effects in patients receiving, for example, cancer- or HIV-chemotherapy [33, 34]. The therapeutic index of delta 9-THC, however, is narrow. Therapeutic efficient plasma THC-levels are often accompanied by the typical cannabinoid side effects and these may be indicated in special populations, such as chemotherapy-induced nausea or cachexia. By contrast, possibly due to the narrow therapeutic index [15], conflicting reports have emerged upon the clinical use of delta 9-THC in different areas of pain therapy [14] and cannabinoids were acknowledged as coanalgesic adjunct rather than a pure analgesic [21–23, 35]. Also, delta 9-THC should be considered as a psychopharmacological analgesic medication [20]; therefore a careful risk/benefit analysis of cannabinoid treatment may be required, including the evaluation of misuse risk assessment of patients addiction behaviour [20] and withdrawal strategies in cases misuse occur.

 In the present retrospective survey we assessed delta 9-THC therapy in central neuropathic pain and fibromyalgia patients. Even though it appears premature to classify fibromyalgia as a neuropathic pain syndrome [29], these patients suffer chronic pain and often from an impaired quality of life due to depression, sleep deprivation, or other functional somatic syndromes. Therefore, these patients may profit from a coanalgesic and psychopharmacologic delta 9-THC therapy. About 25% of patients, however, withdrew from delta 9-THC administration for various reasons, among others due to a self-assessed ineffective therapy. Those patients who did respond to delta 9-THC therapy, the vast majority reported a significant reduction of pain intensity, a relevant improvement of mood and quality of life, and a lowering of concomitant opioid medication. 

### 4.1. Pain Intensity

 In our patient sample, THC treatment led to a significant reduction in pain intensity. Noteworthy, this effect could be observed when a mean daily dose of 7.5 mg THC was administered. This dosage shows high acceptance and efficacy.

 According to our findings, positive reports had been reported previously in smaller patient samples in which delta 9-THC was used for therapy of refractory neuropathic pain states [16]. The authors described the combination of cannabinol and cannabidiol being effective in the treatment of central neuropathic pain in multiple sclerosis patients. A recent meta-analysis supported these findings [36]. In addition, other clinical trials in which central neuropathic pain patients were treated with cannabinoids revealed a modest but clinically relevant analgesic effect in the treatment group when compared to placebo [37, 38]. In addition, in the present survey, patients reported that efficacy and acceptance of therapy were significantly better during THC treatment. It should be noted that a cannabinoid-based analgesia is not for acute or subacute pain therapy. However, after a beneficial trial, THC may be considered as a longer term coanalgesic. As reported previously after oromucosal cannabis-based therapy of central neuropathic pain patients, the perceived pain intensity was reduced and sleep disturbances improved over a time period exceeding 1 year [39, 40].

 No effects of delta 9-THC, however, were observed in postoperative pain management [41] and experimental human pain models [23]. Also, a study conducted in refractory central neuropathic pain patients did not support an overall benefit of THC on pain and quality of life upon sublingual administration of the cannabinoid nabilone [42]. In this investigation, however, treatment was terminated in 5 out of 7 patients due to intolerable side effects, probably caused by the administered doses of up to 25 mg/day. The short period of drug administration in these studies may explain a lack of analgesic effect. If a single dose exceeds 20 mg THC, side effects are likely to dominate before the appearance of pharmacological effects. THC is lipophilic and has a complex central pharmacokinetic with unexpectedly long half life in the CNS. Therefore, as reported by Rog and colleagues, an efficient cannabinoid therapy may be achieved by long-term administration of delta 9-THC at low doses [39] to enable a steady-state tissue THC-concentration, which also would meet the narrow therapeutic range of THC as a psychogenic drug.

### 4.2. Quality of Life

A further important and beneficial effect of delta 9-THC therapy is the change of the patient's mood that can occur in addition to pain reduction. Animal data suggested an antidepressant effect of THC [43, 44], which is supported by the present survey showing a significant reduction of depression in the patients during treatment. This effect likely is attributed to an activation of cannabinoid receptors in the brain. In human neocortex and amygdala, the CB1 receptor is frequently expressed. The amygdala, the anterior cingulate cortex, and the prefrontal cortex are key structures in the brain for memory, for the perception and emotional processing of pain, and also for the integration of mood modulation. In an animal model, Marsicano et al. demonstrated that aversive memory can be extinguished after application of THC [45]. Also, in experimental catastrophic situations, THC was able to diminish stress reaction in animals [43, 44]. Given that pain memory is a crucial mechanism of maintaining pain perception, these experimental data may be of importance to explain the beneficial effect of delta 9-THC in the treatment of chronic pain patients. The intake of antidepressants and anticonvulsants in neuropathic pain states has been linked to pain memory and mood changes. Their effectiveness apparently increased during THC-therapy, indicated by a reduced administration. Thus, therapeutic effects of delta 9-THC might be based, at least to some extent, on pain memory extinction and mood changes.

 Importantly, in this context, delta 9-THC treatment additionally improved health-related quality of life, as indicated here by the PDI and SF12 scores. Previous data recorded in humans support this finding [2, 46]. Particularly, patients suffering multiple sclerosis [37] or pain from brachial plexus avulsion [46] appraise THC therapy and report better quality of life. To assess the effect of therapeutic intervention, however, pain research has mainly focused on the reduction of pain intensity, even though quality of life parameters may reflect clinical improvement for the patients much better. In this respect, ability to work or job impairment is important factors. Here, we found that work-related situations improved after THC treatment. Data on this topic with respect to central neuropathic pain and fibromyalgia are missing so far, as most studies investigating environmental aspects improving work-life refer to musculoskeletal pain [47].

### 4.3. Concomitant Medication

Administration of delta 9-THC supplemental to the current pain medication did not deteriorate therapy; rather patients were able to reduce the analgesics, particularly the intake of opioids. An interaction between cannabinoids and opioids has been reported in previous experimental studies before and it was suggested that delta 9-THC induced effects are mediated also through delta and kappa opioid receptors [48]. Interestingly, a reciprocal alteration of receptor density has been observed in presence of cannabinoids and opioids [49]. This observation also may include modified receptor activation, for instance, delta 9-THC enhanced opioid receptor recruitment, which would explain a reduced opioid medication. Thus, as suggested recently [50], drug combinations should be considered for therapy, and as presented here, delta 9-THC may represent one option of medication. 

 Intriguingly, of the concomitantly administered medication, no change in the use of NMDA antagonists was recorded. This observation might be attributed to a lacking additional effect of cannabinoids with NMDA antagonists. Also, an inhibitory action of selective NMDA antagonists on the antinociceptive efficacy of cannabinoids was reported recently in the rat periaqueductal grey [51], which may require an increased cannabinoid administration rather than a reduced NMDA antagonist medication for therapy.

### 4.4. Limitations

A limitation of the study is performance of a *single* telephone survey by one interviewer. A face-to-face *serial* interviews may be suggested as a better study design alternative, which was considered during development of the study protocol. Performing a multicenter study would require a large number of interviewers conducting the face-to-face interview. The parameter assessment by different interviewers may cause variations, particularly considering psychometric parameters, and irrespective the experiences of the interviewers. A telephone survey, performed by one interviewer only, may be minimally influenced by external factors and therefore suggests consictency of the evaluated parameters in the patient. 

 Another limitation may be due to the heterogenous patient group with very little selection criteria. This justifiable concern actually was one objective of the survey, that is, to explore the applicability and usefulness of an additional coanalgesic cannabinoid treatment in a broad and unspecific group of pain patients. Consequently, the relatively high drop out rate of about 25% may be due to the virtually absent selection criteria of the patients, but also may have caused by the narrow therapeutic index of THC, as mentioned previously.

 Amelioration of pain and quality of life improvement were major outcome parameters. In future surveys, therefore, further limiting factors like practice variation, duration of medication, assessment of addiction behaviour, and misuse risk should be considered and included in future interview protocols.

## 5. Conclusions

Patients taking delta 9-THC for pain therapy can be interviewed easily by phone to explore their tolerance towards the medication. Some but not all patients who respond and tolerate delta 9-THC administration may benefit considerably from this coanalgesic. Acceptance and tolerance of a short-lasting delta 9-THC trial therefore would determine a selection criterion for an additional treatment option to reduce pain, to decrease the comedication, and to improve quality of life. Transient delta 9-THC titration up to about 15 mg, its continued administration for a few weeks, the documentation of the patients tolerance to the therapy with putative side effects, and the recording of the patients' self-reported pain estimates would reveal the efficacy and tolerability of a supplemental cannabinoid-based coanalgesic medication.

## Figures and Tables

**Table 1:** Etiology of the diagnosed central neuropathic pain. Neuropathies within the patient cohort were grouped by an inflammatory origin (*n* = 43) or a trauma of the central nervous system (*n* = 49). Note that fibromyalgia patients (*n* = 32) are not listed.

Inflammatory	Central neuropathy
central neuropathy	due to trauma
Multiple sclerosis (*n* = 32)	Paraplegia (*n* = 8)
Encephalitis (*n* = 9)	Stroke (*n* = 10)
Others (*n* = 2)	Intracranial injury (*n* = 4)
	Neoplasm (*n* = 4)
	Others (*n* = 23)

**Table 2:** Medication administered to the patients before and during/after delta 9-THC therapy. A substantial reduction of the medication within each group, that is, nonopioids, opioids, or nonanalgesics (primarily antidepressants and anticonvulsants), could be recorded during the treatment with delta 9-THC.

	Before delta 9-THC therapy			During/after delta 9-THC therapy	
Comedication		Number of patients	%	Number of patients	%
Nonopioids	NSAID	60	48	7	5.6
	COX2-inhibitors	34	27	7	5.6
	Paracetamol	29	23	3	2.4
	Metamizole	44	35	10	8.1
	Flupirtin	37	30	7	5.6
	others	14	11	2	1.6

Opioids	Tramadol	35	28	2	1.6
	Naloxone	36	29	5	4
	Buprenorphin/fentanyl	15	12	5	4
	Morphin	22	18	7	5.6
	Hydromorphone	17	14	10	8.1
	Oxycodone	13	10	5	4
	Others	6	5	5	4

Nonanalgesics	Antidepressants	68	55	18	14.5
	Anticonvulsants	40	32	18	14.5
	Corticosteroids	19	15	6	4.8
	NMDA-antagonists	5	4	5	4
	Others	21	17	7	5.6

**Table 3:** Estimated pain intensity (VRS) and maximum/minimum pain (NRS) recorded in the subgroups of “inflammatory central neuropathy”—“central neuropathic pain due to trauma”—“fibromyalgia” prior to and during/after delta 9-THC therapy. No significant differences between the groups could be analysed. In each group, delta 9-THC medication caused a noticeable amelioration of pain.

	Inflammatory central neuropathy		Central neuropathy due to trauma		Fibromyalgia	
	Prior to	After	Prior to	After	Prior to	After
	Delta 9-THC medication		Delta 9-THC medication		Delta 9-THC medication	
Pain intensity	7.6 ± 1.7	4.2 ± 1.9	7.6 ± 1.4	3.8 ± 1.5	7.9 ± 1.5	4.4 ± 1.5
(VRS, 0–10)						
Max. pain	7.6 ± 1.7	4.9 ± 2.4	9.3 ± 1	5.3 ± 1.7	9.3 ± 1.1	6.1 ± 2.1
(NRS, 0–10)						
Min. pain	5.4 ± 1.8	2.1 ± 1.5	6.0 ± 1	2.9 ± 1.9	6.6 ± 2	3.0 ± 1.8
(NRS, 0–10)						
